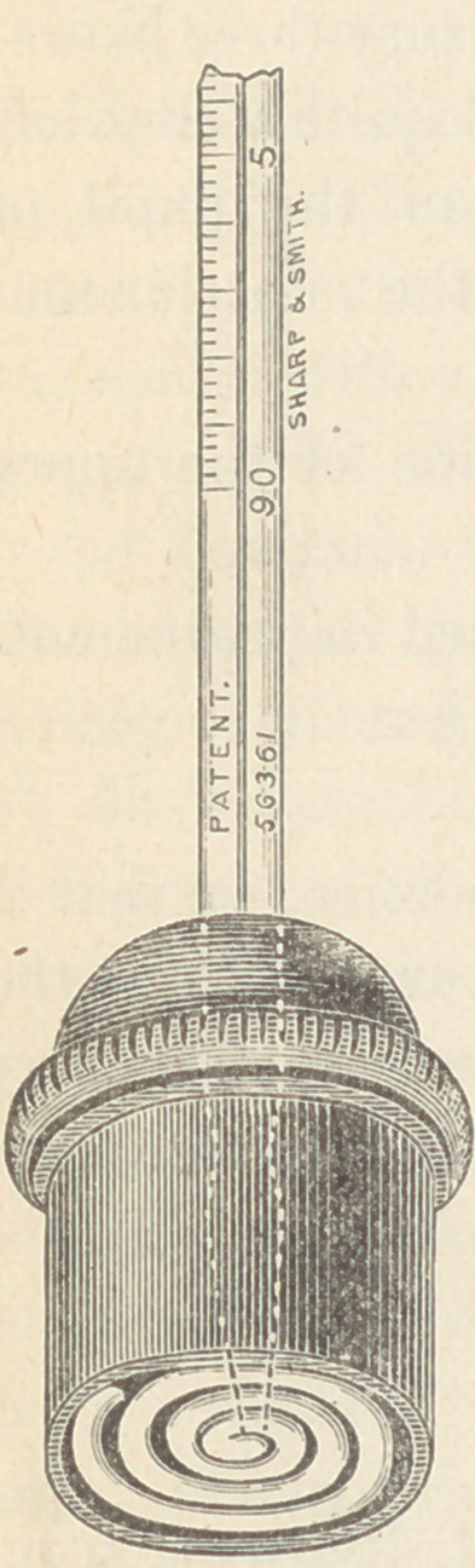# A New Surface Thermometer

**Published:** 1880-05

**Authors:** Daniel R. Brower

**Affiliations:** Chicago


### ARTICLE VI.

### A NEW SURFACE THERMOMETER. By DANIEL R. BROWER, M.D., Chicago.

That the surface temperature varies with the change in the nutrition of the parts beneath, is a fact beyond question, and if we can establish a standard of variation for different disturbances of nutrition, we will have in this a valuable aid to diagnosis. To make these observations useful to the busy practitioner the instrument used for the purpose must make rapid and accurate records.

The original surface thermometer as devised by Dr. Seguin is unreliable because, as is well known, a moderate pressure, made upon the bulb will alter the height of the column of mercury one or two degrees—the maker aiming at rapidity, sacrificed accuracy. Dr. L. C. Gray obviated this difficulty by having the bulb made so heavy that ordinary pressure would make but little alteration in the record—but this extra thickness of the glass makes a much longer time, necessary for a correct observation. This instrument should remain in situ about fifteen minutes.

A new instrument was therefore demanded with which an accurate observation could be made in a shorter time, and this seems to be well accomplished in the thermometer, recently sent us by Messrs. Sharp & Smith of this city, made by Hicks, of London.

It is well represented in the accompanying cut. The bulb is peculiar in its construction. Instead of being flat, as in the Seguin instrument, or elongated as in the ordinary one, it is made of a slender glass tube coiled upon itself three times—exposing a large surface of mercury to the body in such a shape that pressure makes no impression upon the column. The stem has a contracture as in the ordinary thermometer to prevent loss of the index. This stem passes tightly through a rubber diaphragm that serves to keep the coil closely applied to the surface The coil and diaphragm are enclosed in a hard rubber case that effectually guards the former from varying conditions of external temperature and serves to give the hand a firm grasp of the instrument while being applied.

We have found after numerous tests that this instrument will not only give an accurate record of temperature but will give it in one-half the time the Gray instrument requires.

## Figures and Tables

**Figure f1:**